# Tardiness hair in transitional zone: Analysis between age and frontal forehead hairline in children aged below 5 years old

**DOI:** 10.1371/journal.pone.0336923

**Published:** 2025-12-04

**Authors:** Wenting Wu, Ye Liu, Yu Zhang, Tingting Li, Yuehua Men

**Affiliations:** 1 Department of Dermatology, Peking University Third Hospital, Beijing, China; 2 Department of Endocrinology, Peking University Third Hospital, Beijing, China; Faculty of Medical Sciences of Minas Gerais, BRAZIL

## Abstract

**Objective:**

This study aimed to investigate the morphological characteristics and distribution of frontal hairline patterns among Chinese infants and young children aged 0–5 years and to explore the correlation between age and hairline morphology.

**Methods:**

A cross-sectional study was conducted on 213 infants and young children aged 0–5 years in the Dermatology Department of the Third Hospital of Peking University between February 2017 and February 2023. Participants are divided into four types based on the angle formed between the line connecting the point at the frontotemporal angle of the hairline and the tragus and the coronal plane[greater than 30 degrees (Type I), 15–30 degrees (Type II), 0–15 degrees (Type III), and less than 0 degrees (Type IV)]. The presence of temporal peak and skin color phototypes were also recorded. Ordinal logistic regression and restricted cubic splines were used to analyze the relationship between age and hairline phenotype.

**Results:**

Among the 213 participants, Type II (40.8%) and Type III (39.4%) hairlines were the most prevalent, while Type I (6.6%) was the least common. Multivariate regression analysis revealed a negative correlation between age and hairline type, which persisted after adjusting for potential confounding factors such as gender, temporal bulge, and skin phototype. Restricted cubic spline analysis further revealed a non-linear, inverse J-shaped relationship between age and hairline type.

**Conclusion:**

In infants and young children aged 0–5 years old, Type II and Type III frontal hairline patterns were most relevant. A negative correlation between age and hairline type were observed in this population.

## 1. Introduction

The hairline, which delineates the boundary between the frontal eminence and the pilosebaceous follicles, exhibits a unique configuration for each individual, akin to dermal ridge patterns [1]. The morphological attributes and vertical positioning of the hairline are crucial determinants of facial aesthetics, warranting substantial investigation within the field of craniofacial aesthetics [2].

Historically, research on the hairline has primarily focused on its alterations and degeneration caused by dermatologic and trichological pathologies, such as androgenetic alopecia, traction alopecia, and frontal fibrosing alopecia [3]. Empirical data have consistently shown that, regardless of gender, the hairline tends to stabilize or gradually recede post-adolescence, evolving from a concave configuration during early childhood (around the age of five) to the typical convex or M-shaped pattern observed in adult males [4]. In adult females, the hairline is generally characterized by a stable, planar contour [2].

Traditional hairline assessment methods rely on anatomical landmarks including the midline frontal point, widow’s peak, frontotemporal recess, temporal mound, and temporal peak, as described by Shapiro & Shapiro [5]. However, these landmarks are developmentally absent in children under 5 years of age, as demonstrated by Rassman et al. [6] who studied 1,051 children and found that “the hairline is always a continuous and smooth circular shape” without traditional landmarks. This fundamental developmental difference necessitates alternative assessment approaches for pediatric populations. Angular measurements have demonstrated superior reliability in pediatric craniofacial assessment, with intraclass correlation coefficients consistently >0.88, compared to subjective landmark identification which shows only fair to moderate agreement (ICC 0.62–0.84) in young children [7,8].

However, there is a paucity of descriptive and experimental research on hairline morphology in children under the age of 5 years old. The study by William et al. was limited to observing hairline phenotypes in individuals above this age threshold [4]. Observations from daily life, social interactions, and media suggest that infants’ hairlines often resemble the M-shaped male pattern, with more pronounced recession at the temporal regions compared to the mid-forehead, resulting in a broader convex silhouette that may include a widow’s peak.

Furthermore, incomplete follicularis maturation in infancy leads to a more extensive transition from vellus to terminal hair, or to a denser follicular unit terminal hair, producing a hairline phenotype that resembles those seen in some adult males with androgenetic alopecia [9,10]. The present study aims to corroborate the prevalence of these observed characteristics among young children and to investigate the factors influencing the development of the hairline phenotype in this population. Additionally, this research seeks to establish a foundation for future studies examining the evolution of hairlines from infancy to adulthood within the Chinese population.

## 2. Methods

### 2.1 Study population

Patients in this study was derived from patients aged 0–5 years old who visited the Dermatology Department of the Third Hospital of Peking University between February 2017 and February 2023. Data were accessed for research purposes on 24/08/2024. The authors had no access to information that could identify individual participants during or after data collection. Patients who had frontal facial photographs taken without headwear for treatment purposes in our department were included. All patients and their guardians were informed about the study and signed written informed consent forms.

### 2.2 Indicators measurements

**Fig 1:**
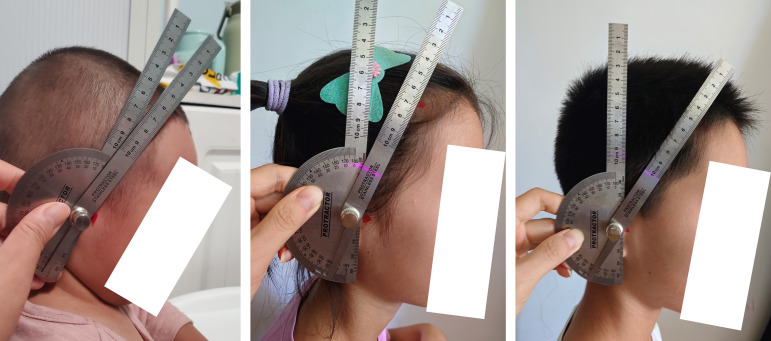
Angular measurement methodology in pediatric subjects.

Participants are divided into four types based on the angle formed between the line connecting the point at the frontotemporal angle of the hairline and the tragus and the coronal plane[greater than 30 degrees (Type I), 15–30 degrees (Type II), 0–15 degrees (Type III), and less than 0 degrees (Type IV)]. To ensure measurement accuracy and eliminate potential photographic distortion errors, angular measurements were performed using direct protractor application rather than photographic analysis. As demonstrated in Fig 1, a standard geometric protractor was placed directly against the participant’s head to measure the frontotemporal angle. This direct measurement approach ensures standardization and reproducibility while minimizing positioning artifacts that could occur with photograph-based assessment methods. All measurements were conducted by trained personnel using identical protractor instruments to maintain consistency across the study population.

Skin color phototype was documented according to the Fitzpatrick classification system based on the photographs: Type I: Pale skin that is prone to sunburn and does not tan; Type II: Fair skin that burns easily and tans poorly; Type III: Light brown skin that sometimes burns and tans progressively; Type IV: Brown skin that rarely burns and tans easily; Type V: Dark brown skin that is less likely to burn and tans darkly; Type VI: Black skin that never burns and tans very easily.

### 2.3 Sample size calculation

This study was a cross-sectional study, and the sample size was calculated using the formula n=[Z²_1-α/2_ × p × (1-p)]/δ². With a significance level α = 0.05, the test statistic Z1-α/2 was 1.96. Based on a preliminary survey, the incidence of hairline recession in children was found to be p = 0.9, and the permissible error δ = 0.05 × p = 0.045. The result indicated a required sample size of 171. To ensure sufficient statistical power, the sample size was increased by 25%, resulting in a final sample size of n = 214. A simple random sampling method was used to select participants.

### 2.4 Statistical methods

Continuous variables were expressed as mean ± standard deviation (SD) or median (P25, P75), while categorical variables were presented as percentages or proportions. For multivariate analysis, ordinal logistic regression was employed to analyze the differences in hairline patterns across age groups. To account for potential non-linear trends, restricted cubic splines (RCS) were applied to analyze the correlation between age and hairline morphology. Statistical significance was defined as P < 0.05. Data analysis was conducted using SPSS software version 27.0 (IBM Corp., Armonk, NY, USA) and R version 4.4.0 (http://www.R-project.org).

## 3. Results

### 3.1 Baseline characteristics

**Fig 2:**
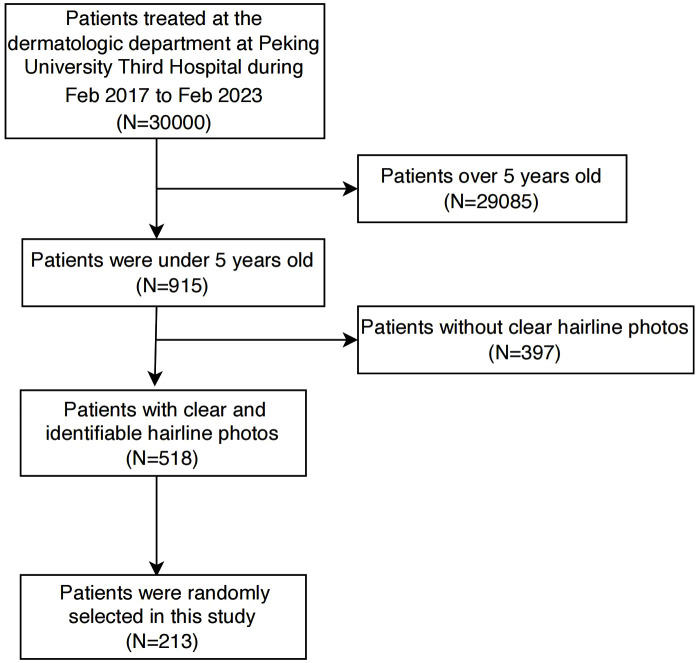
Patients flowchart.

**Fig 3:**
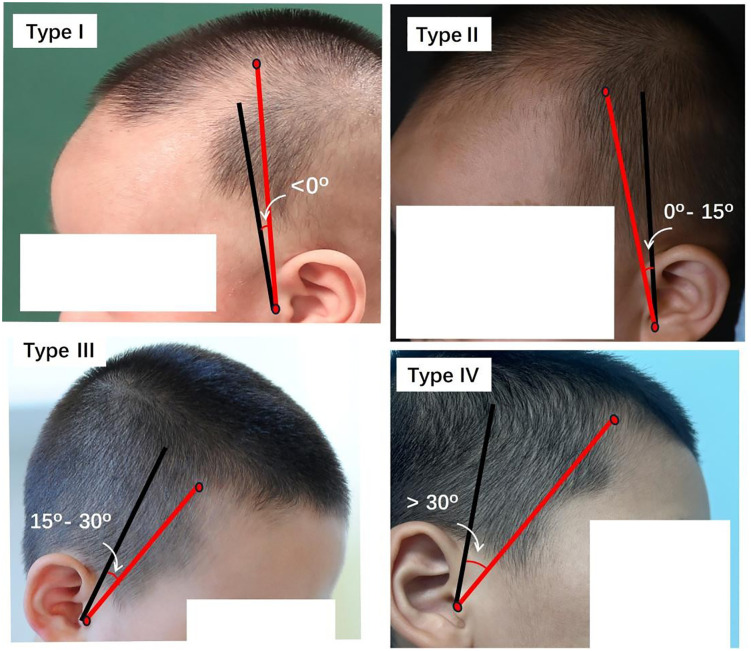
Images of the hairline of patients classified from Type I to Type IV.

Between February 2017 and February 2023, a total of 30000 patients were treated at the dermatologic department of Peking University Third Hospital. Among them, 518 individuals had clear and identifiable hairline photos without headwear, and 397 met the inclusion criteria. After random sampling, a final cohort of 213 patients was enrolled. The patient flowchart was detailed in Fig 2. Fig 3 presented a detailed representation of the hairlines for patients with Type I to Type IV.

Of the enrolled patients, there were 99 males and 114 females with an average age of 1.8 ± 1.7 years. The age distribution was as follows: 64 children aged 0 to less than 1 year, 48 children aged 1 to less than 2 years, 33 children aged 2 to less than 3 years, 26 children aged 3 to less than 4 years, 18 children aged 4 to less than 5 years, and 24 children aged 5 to less than 6 years. Among all patients, Types II and III were the most prevalent, accounting for 40.8% and 39.4% of the cases, respectively, while Type 1 was the least common, at 6.6%. Of the entire patient cohort, 145 individuals had temporal peak, representing 68.1% of the total. Regarding the distribution of skin phototypes, Types II and III were again the most frequent, comprising 45.5% and 42.3% of the cases, respectively, with Type IV being the least prevalent at 3.3%. The details of the distribution were presented in Table 1.

**Table 1:** Baseline characteristics of the enrolled patients.

Variables	Numbers	Proportions (%)
Age (year)		
0	64	30.0
1	48	22.5
2	33	15.5
3	26	12.2
4	18	8.5
5	24	11.3
Sex		
Male	99	46.5
Female	114	53.5
Hairline recession types		
Type I	14	6.6
Type II	87	40.8
Type III	84	39.4
Type IV	28	13.1
Temporal Peak		
Yes	145	68.1
No	68	31.9
Skin Color		
Type I	19	8.9
Type II	97	45.5
Type III	90	42.3
Type IV	7	3.3

### 3.2 Hairline patterns by age group

**Fig 4:**
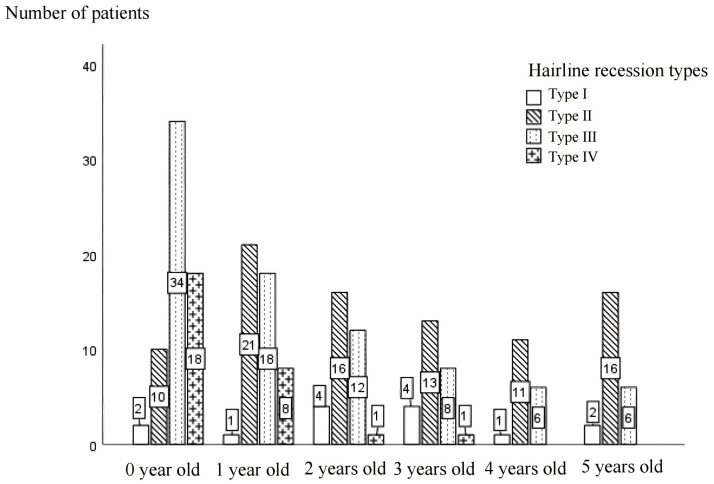
Morphological composition of hairline in children of different ages.

As depicted in Fig 4, the distribution of hairline pattern types varies across different age groups. In infants aged 0–1 year old, Type III hairline patterns are the most prevalent, and their proportion gradually decreases with increasing age.

### 3.3 Multivariate regression of the correlation between age and hairline pattern

Multivariate regression analysis was performed to investigate the correlation between age and hairline pattern, as shown in Table 2. Three models were constructed, with age treated as either an ordinal polytomous variable or a continuous variable. In Model 1, infants aged 0 years had a significantly higher odds ratio (OR) of 10.70 (95% CI: 4.17–27.46, P < 0.01) for higher hairline types (e.g., Type IV) compared to the reference group of 5-year-olds. Model 2 yielded similar results, with 0-year-olds (OR=10.72, 95% CI: 4.17–27.58, P < 0.001) and 1-year-olds (OR=3.62, 95% CI: 1.41–9.32, P = 0.008) showing significantly higher odds of higher hairline types. In Model 3, after adjusting for gender, temporal peak, and skin phototype, 0-year-olds (OR=9.73, 95% CI: 3.57–26.52, P < 0.001) and 1-year-olds (OR=3.26, 95% CI: 1.24–8.55, P = 0.02) still exhibited significantly higher odds of higher hairline types compared to 5-year-olds.

**Table 2:** Multivariate regression of the correlation between age and hairline pattern.

	Model 1		Model 2		Model 3	
	OR (95%CI)	P value	OR (95%CI)	P value	OR (95%CI)	P value
0 year old	10.70 (4.17-27.46)	<0.01	10.72 (4.17-27.58)	<0.001	9.73 (3.57-26.52)	<0.001
1 year old	3.62 (1.41-9.32)	0.008	3.62 (1.41-9.32)	0.008	3.26 (1.24-8.55)	0.02
2 years old	1.47 (0.54-4.03)	0.45	1.47 (0.54-4.03)	0.45	1.50 (0.54-4.21)	0.44
3 years old	1.172 (0.40-3.43)	0.77	1.175 (0.40-3.45)	0.77	1.08 (0.37-3.18)	0.89
4 years old	1.37 (0.44-4.30)	0.59	1.37 (0.44-4.30)	0.59	1.32 (0.41-4.24)	0.64
5 years old	1 (Ref)	NA	1 (Ref)	NA	1 (Ref)	NA
OR for trend	0.83 (0.78-0.88)		0.62 (0.52-0.73)		0.64 (0.53-0.76)	
P for trend	<0.001		<0.001		<0.001	

OR: odds ratios.

Model 1: No adjustment for any potential confounders.

Model 2: Adjusted for sex.

Model 3: Adjusted for sex, skin color, and temporal peak.

When age was treated as a continuous variable, a significant negative correlation between age and hairline pattern was observed across all three models. The OR for trend was 0.83 (95% CI: 0.78–0.88, P < 0.001) in Model 1, 0.62 (95% CI: 0.52–0.73, P < 0.001) in Model 2, and 0.64 (95% CI: 0.53–0.76, P < 0.001) in Model 3, indicating that younger ages were associated with a higher likelihood of higher hairline types, even after controlling for potential confounding factors.

### 3.4 Restricted cubic spline analysis

**Fig 5:**
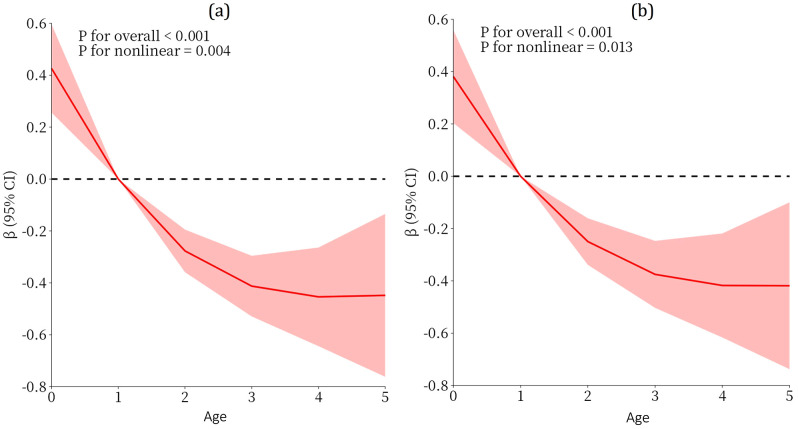
Restricted cubic splines for age and hairline type: (a)unadjusted; (b) adjusted for Adjusted for sex, skin color, and temporal peak.

As illustrated in Fig 5a and 5b, a non-linear trend analysis between age and hairline pattern was conducted using Restricted Cubic Splines (RCS) analysis. After treating the hairline pattern as a continuous variable, both univariate and multivariate analyses revealed a non-linear relationship between age and hairline pattern, which was statistically significant. The graphical representation exhibits an inverse J-shaped correlation, indicating a negative association.

## 4. Discussion

Our study showed that Type II (40.8%) and Type III (39.4%) hairlines accounted for the majority, while Type I (6.6%) was the least common in children aged below 5 years old. Multivariate regression analysis revealed a negative correlation between age and hairline type, which persisted even after adjusting for potential confounding factors such as gender, temporal peak, and skin phototype.

The frontal hairline is a crucial anatomical landmark and aesthetic feature of the face, playing a significant role in an individual’s appearance and self-perception. Numerous studies have investigated the characteristics and progression of the frontal hairline in adults, particularly in the context of androgenetic alopecia. A seminal study investigated the hairline patterns of men and women aged 18–50 years and proposed a classification system based on the shape and position of the hairline [2]. They found that most men had an M-shaped or V-shaped hairline, while women predominantly exhibited a round or oval hairline. Subsequent studies by Rassman et al. and Gupta et al. further refined the classification of male and female hairline patterns, respectively, and explored the relationship between hairline morphology and age [4,11].

In the pediatric population, research on the morphology and development of the hairline in infants and young children remains limited. A study examined the hairline patterns of African children aged 5–15 years and found that the majority had a round or oval hairline, with no significant differences between boys and girls [12]. However, this study did not include children under the age of 5 years. Other studies have explored the potential genetic and environmental factors influencing hairline morphology. A study found a significant genetic component to male-pattern baldness, which is characterized by a receding frontal hairline. The authors identified several genetic loci associated with the condition, suggesting that hairline morphology may be influenced by complex genetic factors [13]. Similarly, a study by Chumlea et al. found that the prevalence of male-pattern baldness varied across different ethnic groups, with the highest rates observed in Caucasians and the lowest in African Americans, indicating that genetic and racial factors may play a role in hairline morphology [14].Environmental factors, such as diet and lifestyle, have also been implicated in the development and progression of hairline recession. A study found that a Mediterranean diet, rich in fruits, vegetables, and olive oil, was associated with a lower risk of androgenetic alopecia in men [15]. The authors suggested that the antioxidant and anti-inflammatory properties of these foods may help to protect against hair loss and promote healthy hair growth. Similarly, a study by Severi et al. found that smoking and high body mass index were associated with an increased risk of male-pattern baldness, suggesting that lifestyle factors may influence hairline morphology [16].

While these studies have provided valuable insights into the factors influencing hairline morphology in adults and older children, there remains a paucity of research on the characteristics and development of the hairline in infants and young children. Contrary to the pattern observed in older populations, where hairlines gradually recede with age, we found that the hairline in infants and young children aged 0–5 years tends to advance with increasing age. We identified a ‘delayed’ phenomenon in the development of certain parts of the frontal hairline. Previous studies found an ‘early retreat’ phenomenon in these areas, especially in the temporal mound and the regions on both sides of the frontal widow’s peak [2]. In most newborns, the hairline is far behind the mature hairline at birth, and these areas of hair, which only grow in after birth, are also the first to be affected in physiological recession or androgenetic alopecia in adults [9,17]. We speculate that the physiological backward movement of the hairline with increasing age shares overlapping principles and mechanisms with the occurrence of androgenetic alopecia. If we define the region between the hairline at birth and the mature hairline as the ‘transitional zone,’ this area and the hair behind it may have different sensitivities to androgens [18,19]. The consistency between the formation of the frontal hairline in infants and young children and the characteristics of physiological aging provides a valuable research window for exploring the growth and development patterns of hair follicles and the mechanisms of senescence [20,21]. Such investigations in this field may contribute to the discovery of novel interventions aimed at promoting hair health, delaying the aging process, and offering new strategies for the treatment of related conditions. Our angular measurement approach is methodologically sound and supported by extensive literature validating quantitative methods in pediatric populations. Studies consistently demonstrate that objective measurements provide superior diagnostic accuracy compared to subjective pattern recognition, particularly in populations where cooperation and positioning present challenges [8]. The genetic background of our Chinese pediatric population further supports our methodological choice. The EDAR gene variant, present in 93% of Han Chinese, influences hair development patterns and may delay the emergence of traditional hairline landmarks [22,23]. This population-specific consideration validates the use of quantifiable methods that do not rely on potentially absent anatomical features. The clinical applicability of our findings is enhanced by the use of directly obtainable angular measurements, which can be easily replicated in clinical practice without specialized equipment, facilitating early identification of atypical hairline development patterns. No significant differences were observed in hairline between males and females in our study population. In prior observations, male pattern hair loss predominantly involves the temporal, frontal, and vertex scalp regions, characterized by progressive hairline recession, whereas female pattern hair loss typically manifests as diffuse thinning concentrated in the central scalp with relative preservation of the frontal hairline [24]. However, our current study failed to detect significant sex-based differences in hairline patterns, suggesting that gender-related biological influences on hairline morphology may not exert deterministic effects during early life stages.

However, it is important to acknowledge our study has several limitations. Firstly, our study included small sample size, which might result in selecting bias. Secondly, the population included in our study was mostly Han nationality, which might have potential lack of generalizability to other ethnic or racial groups. Finally, our study was a cross-sectional investigation, which lacked longitudinal follow-up on the evolution of the hairline patterns.

In conclusion, our study provided valuable insights into the morphological characteristics and age-related changes of the frontal hairline in Chinese infants and young children aged 0–5 years. A negative correlation between age and hairline type may existed in these population. Future studies should investigate the correlation between early childhood angular measurements and adult hairline patterns, potentially establishing predictive models for hairline maturation. Additionally, validation studies comparing our angular system with traditional methods in populations where both approaches are feasible would further strengthen the evidence base.

## Supporting information

S1 Data Original data. (XLSX)

## References

[1] Natpracha W, Sukanjanapong S, Chanprapaph K, Suchonwanit P. Characterization and classification of different female hairline patterns in the Thai population. J Cosmet Dermatol. 2021;20(3):890–6. doi: 10.1111/jocd.13642 32783356

[2] Nusbaum BP, Fuentefria S. Naturally occurring female hairline patterns. Dermatol Surg. 2009;35(6):907–13. doi: 10.1111/j.1524-4725.2009.01154.x 19397668

[3] Vañó-Galván S, Molina-Ruiz AM, Serrano-Falcón C, Arias-Santiago S, Rodrigues-Barata AR, Garnacho-Saucedo G, et al. Frontal fibrosing alopecia: a multicenter review of 355 patients. J Am Acad Dermatol. 2014;70(4):670–8. doi: 10.1016/j.jaad.2013.12.003 24508293

[4] Rassman WR, Pak JP, Kim J. Phenotype of normal hairline maturation. Facial Plast Surg Clin North Am. 2013;21(3):317–24. doi: 10.1016/j.fsc.2013.04.001 24017973

[5] Shapiro R, Shapiro P. Hairline design and frontal hairline restoration. Facial Plast Surg Clin North Am. 2013;21(3):351–62. doi: 10.1016/j.fsc.2013.06.001 24017977

[6] Rassman WR, Pak JP, Kim J. Hairline evolution as simple as ABC/123. HTFI. 2013;23(6):197–205. doi: 10.33589/23.6.0197

[7] Mercier J-P, Rossi C, Sanchez IN, Renovales ID, Sahagún PM-P, Templier L. Reliability and accuracy of Artificial intelligence-based software for cephalometric diagnosis. A diagnostic study. BMC Oral Health. 2024;24(1):1309. doi: 10.1186/s12903-024-05097-6 39468520 PMC11520516

[8] Jeon S, Lee KC. Comparison of cephalometric measurements between conventional and automatic cephalometric analysis using convolutional neural network. Prog Orthod. 2021;22(1):14. doi: 10.1186/s40510-021-00358-4 34056670 PMC8165048

[9] Whiting DA. Possible mechanisms of miniaturization during androgenetic alopecia or pattern hair loss. J Am Acad Dermatol. 2001;45(3 Suppl):S81-6. doi: 10.1067/mjd.2001.117428 11511857

[10] Ellis JA, Sinclair R, Harrap SB. Androgenetic alopecia: pathogenesis and potential for therapy. Expert Rev Mol Med. 2002;4(22):1–11. doi: 10.1017/S1462399402005112 14585162

[11] Gupta M, Mysore V. Classifications of patterned hair loss: a review. J Cutan Aesthet Surg. 2016;9(1):3–12. doi: 10.4103/0974-2077.178536 27081243 PMC4812885

[12] Khumalo NP, Jessop S, Gumedze F, Ehrlich R. Hairdressing and the prevalence of scalp disease in African adults. Br J Dermatol. 2007;157(5):981–8. doi: 10.1111/j.1365-2133.2007.08146.x 17725667

[13] Brockschmidt FF, Heilmann S, Ellis JA, Eigelshoven S, Hanneken S, Herold C, et al. Susceptibility variants on chromosome 7p21.1 suggest HDAC9 as a new candidate gene for male-pattern baldness. Br J Dermatol. 2011;165(6):1293–302. doi: 10.1111/j.1365-2133.2011.10708.x 22032556

[14] Chumlea WC, Rhodes T, Girman CJ, Johnson-Levonas A, Lilly FRW, Wu R, et al. Family history and risk of hair loss. Dermatology. 2004;209(1):33–9. doi: 10.1159/000078584 15237265

[15] Fortes C, Mastroeni S, Mannooranparampil T, Abeni D, Panebianco A. Mediterranean diet: fresh herbs and fresh vegetables decrease the risk of Androgenetic Alopecia in males. Arch Dermatol Res. 2018;310(1):71–6. doi: 10.1007/s00403-017-1799-z 29181579

[16] Severi G, Sinclair R, Hopper JL, English DR, McCredie MRE, Boyle P, et al. Androgenetic alopecia in men aged 40-69 years: prevalence and risk factors. Br J Dermatol. 2003;149(6):1207–13. doi: 10.1111/j.1365-2133.2003.05565.x 14674898

[17] Stough D, Stenn K, Haber R, Parsley WM, Vogel JE, Whiting DA, et al. Psychological effect, pathophysiology, and management of androgenetic alopecia in men. Mayo Clin Proc. 2005;80(10):1316–22. doi: 10.4065/80.10.1316 16212145

[18] Heilmann-Heimbach S, Hochfeld LM, Henne SK, Nöthen MM. Hormonal regulation in male androgenetic alopecia-Sex hormones and beyond: evidence from recent genetic studies. Exp Dermatol. 2020;29(9):814–27. doi: 10.1111/exd.14130 32946134

[19] Inui S, Itami S. Molecular basis of androgenetic alopecia: from androgen to paracrine mediators through dermal papilla. J Dermatol Sci. 2011;61(1):1–6. doi: 10.1016/j.jdermsci.2010.10.015 21167691

[20] Ge W, Tan S-J, Wang S-H, Li L, Sun X-F, Shen W, et al. Single-cell transcriptome profiling reveals dermal and epithelial cell fate decisions during embryonic hair follicle development. Theranostics. 2020;10(17):7581–98. doi: 10.7150/thno.44306 32685006 PMC7359078

[21] Visscher MO, Hu P, Carr AN, Bascom CC, Isfort RJ, Creswell K, et al. Newborn infant skin gene expression: remarkable differences versus adults. PLoS One. 2021;16(10):e0258554. doi: 10.1371/journal.pone.0258554 34665817 PMC8525758

[22] Mou C, Thomason HA, Willan PM, Clowes C, Harris WE, Drew CF, et al. Enhanced ectodysplasin-A receptor (EDAR) signaling alters multiple fiber characteristics to produce the East Asian hair form. Hum Mutat. 2008;29(12):1405–11. doi: 10.1002/humu.20795 18561327

[23] Fujimoto A, Kimura R, Ohashi J, Omi K, Yuliwulandari R, Batubara L, et al. A scan for genetic determinants of human hair morphology: EDAR is associated with Asian hair thickness. Hum Mol Genet. 2008;17(6):835–43. doi: 10.1093/hmg/ddm355 18065779

[24] Tamashunas NL, Bergfeld WF. Male and female pattern hair loss: treatable and worth treating. Cleve Clin J Med. 2021;88(3):173–82. doi: 10.3949/ccjm.88a.20014 33648970

